# Kawasaki disease: insights into the roles of T cells

**DOI:** 10.3389/fimmu.2025.1582638

**Published:** 2025-07-17

**Authors:** Shuhui Wang, Guanghui Qian, Ying Liu, Xuan Li, Hongbiao Huang, Ling Sun, Haitao Lv

**Affiliations:** ^1^ Department of Cardiology, Children’s Hospital of Soochow University, Suzhou, Jiangsu, China; ^2^ Department of Pediatrics, Institute of Pediatric Research, Children’s Hospital of Soochow University, Suzhou, Jiangsu, China

**Keywords:** Kawasaki disease, systemic immune, T cells, IVIg, Treg - regulatory T cell

## Abstract

Kawasaki disease (KD) is a systemic immune vasculitis characterized by fever and is a common cause of acquired heart disease in children. The etiology of KD remains unclear, but it is generally believed to be an amplified inflammatory cascade caused by the combined action of infection and genetic susceptibility factors. Changes in T lymphocyte subsets and their abnormal activation play an important role in the immune response to KD. This review delves into the critical role of T cells in the pathogenesis of KD, with a particular focus on how the expansion of CD8+ T cells and the imbalance between Th17 and Tregs contribute to IVIG resistance and persistent inflammation. Our analysis suggests that interventions targeting T cell function could potentially improve the clinical prognosis for KD patients. This provides specific directions for future therapeutic strategies, including the use of novel immunomodulatory approaches such as cyclosporine and IL-17/IL-23 inhibitors, aimed at providing new insights into the pathogenesis and treatment of KD.

## 1 Introduction

**Figure 1:**
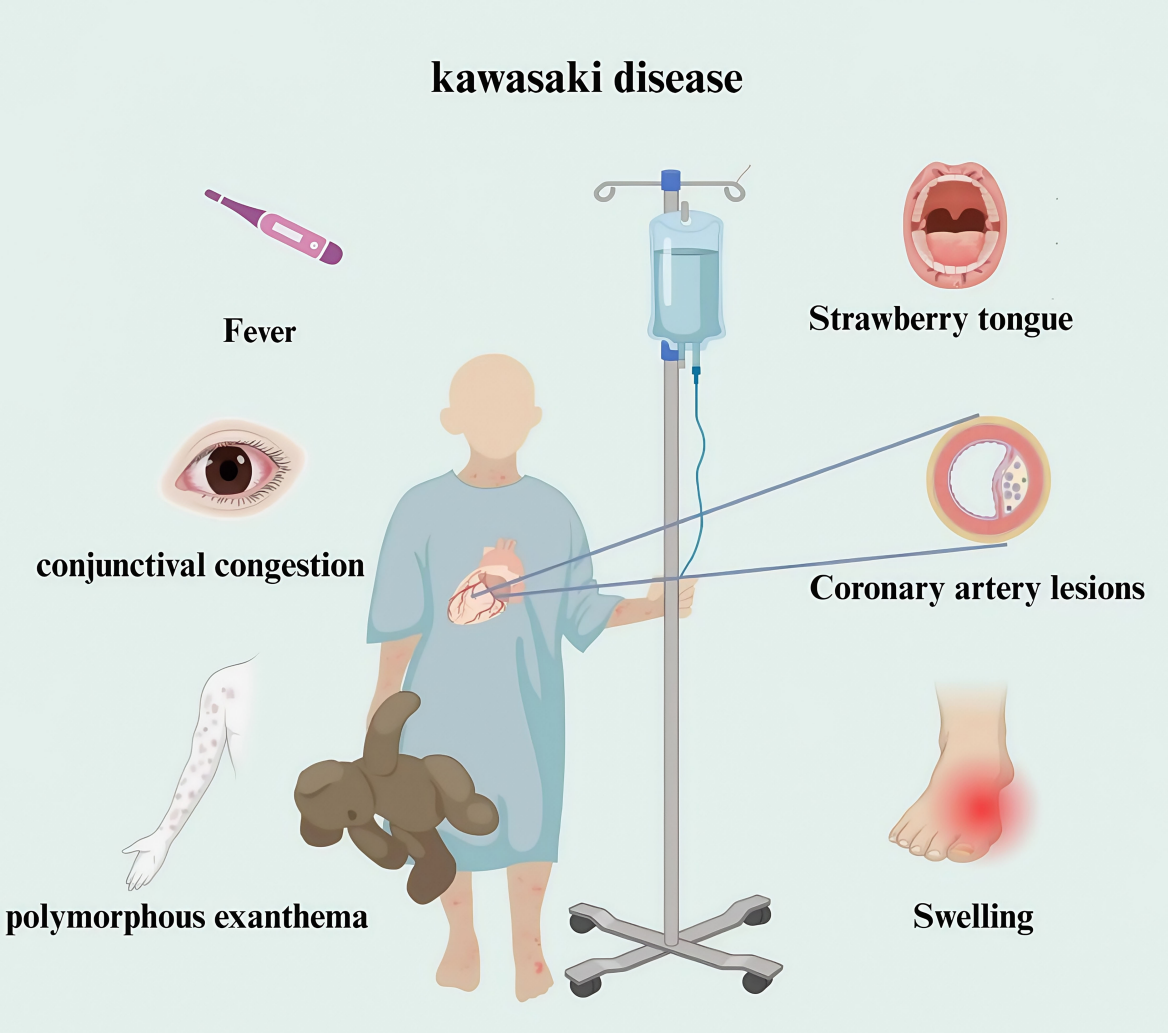
Clinical manifestations of children with Kawasaki disease. Kawasaki disease is a clinical syndrome. The diagnosis mainly depends on clinical manifestations combined with laboratory tests. The main manifestations are fever, accompanied by bilateral bulbar conjunctival hyperemia, strawberry tongue, rash, redness and swelling of hands and feet in acute stage, and coronary artery lesions.

Mucocutaneous lymph node syndrome, commonly known as Kawasaki disease (KD), was reported initially by Dr. Tomisaku Kawasaki in Japan in 1967 (1). KD comprises an acute febrile exanthematous disease characterized primarily by systemic vasculitis. The main clinical features are manifested as fever, bayberry tongue, congestion and chapping of the lips, enlarged cervical lymph nodes, non-suppurative bilateral bulbar conjunctival congestion, rash and swelling of the fingertips which usually occurs in children between 6 months and 4 years of age, and has occasionally been reported in adults (2). It is a global disease with an increasing incidence, and the most frequent underlying source of acquired heart diseases in many developed countries. KD is frequently complicated by serious coronary artery lesions (CALs), If not treated in time, which can have long-term sequelae, including coronary artery occlusion or stenosis (3–6). CALs are the main factor affecting the prognosis of KD and severe cases can be life-threatening because of myocardial infarction (**Figure 1** **).**

T lymphocytes cell (T cells) are hematopoietic stem cells derived from bone marrow. After differentiation and maturation in the thymus, T cells are distributed to the immune organs and tissues of the body through the lymphatic and blood circulation system to exert immune functions. Its dysfunction may lead to excessive immune response, tolerance defect or regulation imbalance, which may lead to a variety of diseases. At present, most studies generally believe that a highly activated immune system, including T cell activation, the release of a large number of inflammatory factors and cascade amplification effects, vascular endothelial dysfunction and immune-injury vasculitis are the significant characteristics of KD. The abnormal activation of T cells is involved in the whole process of inflammatory injury and tissue reconstruction in KD. Overactivated T lymphocytes can produce various cytokines that participate in the inflammatory response and vascular endothelial injury in KD (7–9). At the same time, T cells also have a protective effect by recognizing and clearing pathogens, promoting the immune system to control infection, and facilitating the recovery of vascular endothelial cells. Exploration of the function and regulatory mechanisms of T cells in KD will greatly enhance our understanding of the pathophysiology of KD and the search for more effective treatments. This article reviews the research progress of T lymphocytes in the immune response of KD.

## 2 KD pathogenesis

**Figure 2:**
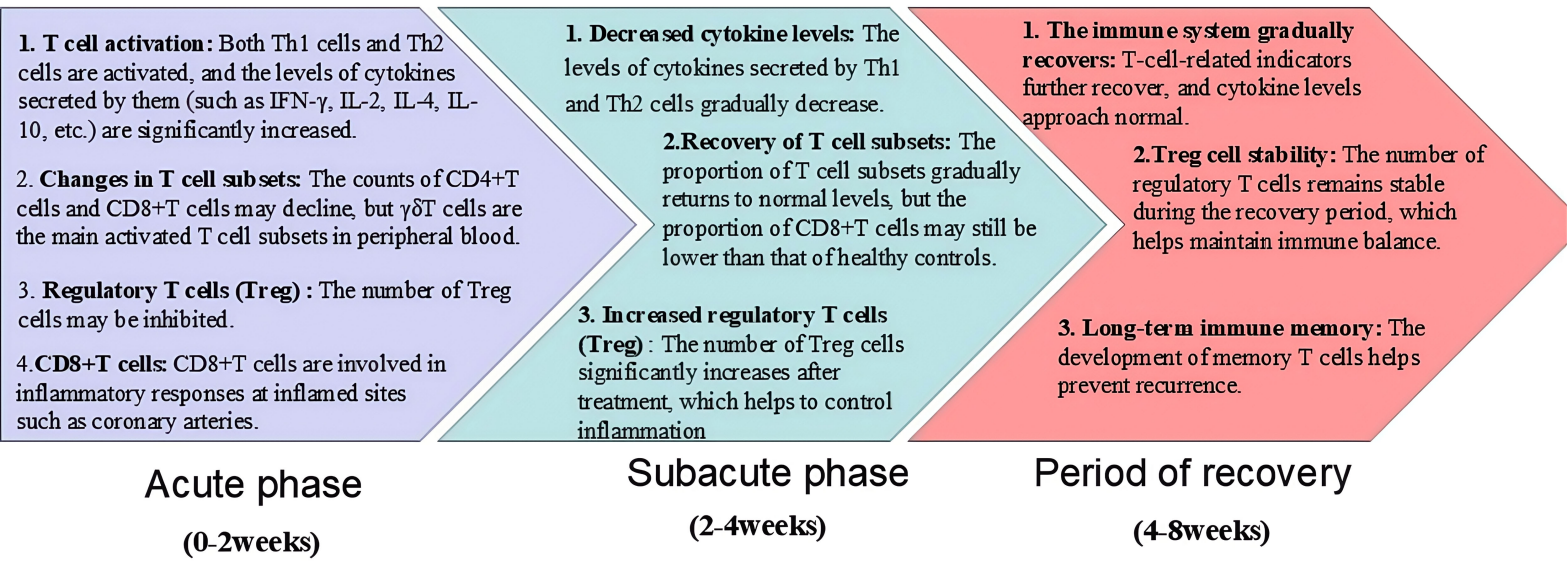
The participation of T cells in different stages of KD (acute phase, subacute phase, and period of recovery).

Orenstein et al. (10) found that the main pathological changes of CAL include self-limited necrotizing vascular arteritis related to neutrophil infiltration in the acute phase, and the pathological changes of CAL include self-limited necrotizing vascular arteritis related to neutrophil infiltration in the acute phase. In the subacute phase, T cells and other inflammatory cells are swollen, necrotic and infiltrated in chronic vasculitis, and in the recovery phase, fibroblast proliferation and thickening damage the coronary artery, and then cause stenosis, occlusion and coronary aneurysm formation (11). As a first-line KD treatment, high-dose intravenous immunoglobulin (IVIG) (2 g/kg) combined with aspirin within 10 days of the onset of KD symptoms is recommended by the American Academy of Pediatrics (AAP) and The American Heart Association (AHA) (5). This treatment regimen can significantly improve the acute symptoms of KD and reduce the incidence of mild CALs. However, the etiology of KD is still unclear. A large number of studies have shown that KD may be caused by microorganisms invading the susceptible body and inducing immune activation, targeting small and medium-sized vessels mainly coronary arteries. The immune imbalance caused by immune activation and then leading to small and medium-sized vascular endothelial inflammation is the main mechanism of CAL in KD (12, 13). Immune imbalance subsequently leads to cytokine storms in various immune cells, especially T cells, causing vascular endothelial edema, elastic fibers and muscle layer rupture, and ultimately resulting in persistent vascular inflammation (14, 15). Studies at home and abroad have confirmed that Endothelial cell dysfunction (ECD) persists for a long time in KD with or without coronary artery dilatation, CAL is closely related to long-term cardiac damage, and abnormal activation of T cells plays an important role in the pathogenesis (7, 16) (**Figure 2**; **Table 1**).

**Table 1:** The key immune mechanism of T cells involved in the occurrence and development of KD and its potential clinical significance.

Title	Role of T cells and their subsets in KD	Potential clinical impact
**Abnormal activation of T cells**	The number of T cells in the peripheral blood of patients with acute KD is decreased, and the proliferation of T cells induced by T cell receptor (TCR) /CD3 is significantly inhibited	1. It may lead to a decrease in the ability of the immune system to clear pathogens and increase the risk of infection. 2. It affects the regulation of inflammatory response and aggravates vascular inflammation.
**Imbalance of T-cell subsets**	**1.Th17 cells**: the proportion of Th17 cells was significantly increased in the acute phase, and oxidized phospholipid and low-density lipoprotein (LDL) could induce the increase of Th17 cells. **2. Regulatory T cells (Treg)** :the proportion of Treg cells decreases in the acute phase, which may lead to excessive inflammatory response. **3.T helper cell (Th) subset:** cTfh1 cells decreased, cTfh2 cells increased, and the ratio of cTfh2 and cTfh17 cells to cTfh1 cells increased	1. The increase of Th17 cells is closely related to the occurrence of coronary artery lesions, which may lead to coronary artery dilatation or coronary aneurysm formation. 2. The decrease of Treg cells may weaken the inhibitory effect on inflammation and aggravate vascular injury. The imbalance of cTfh cell subsets is associated with increased levels of inflammatory markers such as CRP and ESR, which may affect disease severity and treatment response.
**The role of T cells in vascular inflammation**	T cells further recruit immune cells to the vessel wall by secreting cytokines such as IL-1β, TNF-α and chemokines such as CCL2. In animal models, CD8+ T cells are essential for the development of vascular inflammation in Kawasaki disease.	1. Aggravate the inflammatory response of the vascular wall, leading to vascular endothelial cell injury and dysfunction. 2. It may affect the structure and function of coronary arteries and increase the risk of cardiovascular complications.
**Title**	**role of T cells and their subsets in Kawasaki disease**	**Potential clinical impact**
**Differences in response to IVIG treatment**	1. After intravenous immunoglobulin (IVIG) treatment, the level of Treg cells was increased, and the level of Th17 cells was decreased. 2. The failure of IVIG therapy may be related to the abnormal state of T cell subsets and the level of cytokines	1. Monitoring T cell subsets and cytokine levels may help predict treatment response and guide individualized treatment regimens. 2.Other immunomodulatory treatment strategies may need to be explored for patients who do not respond to IVIG therapy.
**Long-term adverse cardiovascular risk**	Even after the acute phase of KD, the patient's immune system may remain in an abnormal state, and the imbalance of T cell subsets may lead to increased cardiovascular risk in the long term	It increases the risk of cardiovascular diseases such as coronary heart disease and myocardial infarction. 2. Long-term cardiovascular follow-up and monitoring of patients with Kawasaki disease are necessary to promptly identify and intervene in potential cardiovascular issues.

## 3 T lymphocyte subpopulation infiltration associated with KD

Mature T cells can be classified into CD4^+^T cells and CD8^+^T cells based on whether they express CD4 or CD8. CD4^+^ T cells are helper T cells, which can assist cellular immunity and humoral immunity. CD8^+^ T cells can inhibit T cell immunity, and effectively fight and clear invading viruses or bacteria. CD4^+^ T cells can assist B cells to produce antibodies and neutralize antigens, and induce and maintain the toxicity of CD8^+^ T cells. Immunohistochemical staining of tissues from deceased pediatric patients with KD revealed infiltration of coronary artery aneurysms by CD4^+^ T cells, CD8^+^ T cells, and CD68^+^ macrophages in patients with acute KD, It was also found that CD8^+^T lymphocytes were 4-5 times more than CD4^+^T lymphocytes in KD vascular lesions. Conversely, for pediatric patients suffering from the acute phase of KD, their peripheral blood exhibited a significant increase in CD4:CD8 ratio, suggesting selective migration of CD8^+^ T lymphocytes from circulation to the primary target tissues in patients with KD (17). Okada et al. (18) found that CD4^+^ T cell were activated in the interstitial lobules of patients with KD, which might be the cause of active antigen presentation and proliferation of reactive lymphocytes in cervical lymphadenopathy. In the mouse model of KD vasculitis induced by the well-recognized *Lactobacillus casei* cell wall extract (LCWE), the vital factor that determined lesion severity was CD8^+^ T cells, and treatment of the model mice with anti-CD8 depletion antibodies prevented the development of vasculitis (19). Thus, CD4^+^ T and CD8^+^ T cells are intimately involved in the KD immune response.

**Figure 3:**
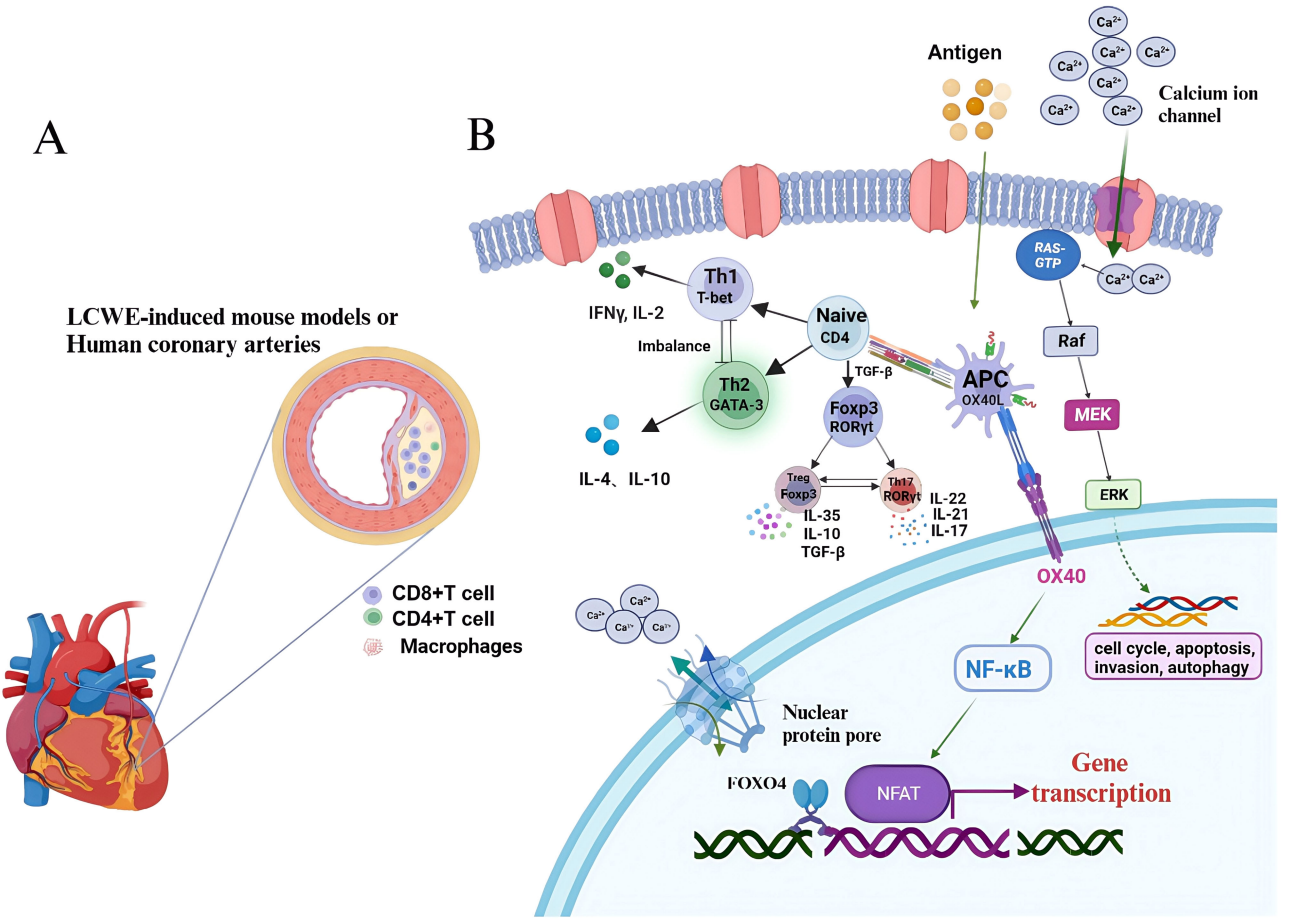
T-cell signaling pathways involved in Kawasaki disease. **(A)** LCWE is injected intraperitoneally into mice to induce coronary arteries, accompanying with infiltrated CD8+ T cells, CD4+ T cells and macrophages. **(B)** T-cell can be activated by DC-presented antigens and differentiate into Th1, Th2, Treg and Th17 via distinct cytokines stimulation. CD4 subsets can involve in the pathogenesis of KD. KD, Kawasaki disease; LCWE, Lactobacillus casei cell wall extract; DC, dendritic cel.

IL-2 is an essential cytokine for the development and function of Tregs, and mice with a deletion of the gene encoding its receptor, IL-2RB, fail to produce a normal subset of Tregs, suggesting that autoimmune diseases are associated with IL-2/IL-2 receptor gene deletion. The presence of the IL-2RA gene with the rs3118470 single nucleotide polymorphism (SNP) combined with the LOC100133214 (rs2517892) SNP increases the risk of CALs in children with KD, and is associated with an increased incidence of KD (20). However, the etiology of KD is still unclear, and most studies proposed that abnormal immune system activation is chiefly responsible for KD vasculitis (21). T cells express high levels of nuclear factor of activated T cells (NFAT), a calcineurin-dependent transcription factor. NFAT has crucial function in T cell activation and also plays an important regulatory role in T cell activation and signal transduction. Bioinformatic analysis showed that the NFAT-mediated signaling pathway interacts with leukocytes to promote the release of inflammatory factors, chemical factors, adhesion molecules, and growth factors, suggesting that NFAT is involved in the activation of T cells and vascular endothelial injury in KD, which might represent an important intermediate link in the pathogenesis of KD (22). In CAWS-induced KD vasculitis mouse model, Forkhead box O4 (FOXO4) can bind to its promoter to inhibit NFAT2 signaling in the NFAT family and attenuate KD vasculitis caused by FOXO4 knockout. FOXO4/NFAT2 axis is involved in the development of KD-related cardiovascular inflammation. Abnormal activation of costimulatory molecules and their ligands, e.g., the OX40 (also known as TNF receptor superfamily member 4)-OX40L (also known as TNF superfamily member 4) signaling pathway, might lead to abnormal activation of NFAT signaling, which leads to KD pathogenesis. Therefore, targeting the OX40-OX40L axis might be a new avenue in the treatment of KD-associated immune injury (22, 23) (**Figure 3**). In addition, Foxp3 directly regulates miR-155, which is highly expressed in Tregs, participating in Treg differentiation, maintenance, or function. Treg downregulation might be related to abnormal miR-155/suppressor of cytokine signaling 1 (SOCS1) signaling and miR-31 overexpression in patients with KD in the acute phase. Treatment with IVIG might regulate miR-155 and miR-31 expression to rescue Treg number and function (24).

Other studies have found that T cells in the peripheral blood of children with KD cannot initiate the normal apoptosis program and continue to proliferate and differentiate, which eventually leads to the increase and abnormal activation of T cells and consequent immune imbalance. In the T cells from children with KD, the activation of extracellular regulated kinase (ERK) by the Raf-MAPK/ERK kinase (MEK)-ERK cascade activates transcription regulators to initiate gene transcription leading to cell proliferation. At the same time, inactivation of the apoptotic gene *P53* and the anti-apoptotic effect of nuclear factor kappa B (NF-κB) P65 result the survival of T cells. However, 1,25(OH)_2_D_3_ can regulate/inhibit excessive T cell proliferation by regulating certain signal transduction pathways (25).

The pathogenesis of KD involves a complex interplay of multiple genes and immune molecules that collectively affect the activation of T lymphocytes, inflammatory responses, and immune regulation. Although research is ongoing, a deeper understanding of these genes and molecules will help to reveal the pathological mechanisms of KD and might provide clues to future therapeutic approaches.

### 3.1 CD4^+^ T cell

**Figure 4:**
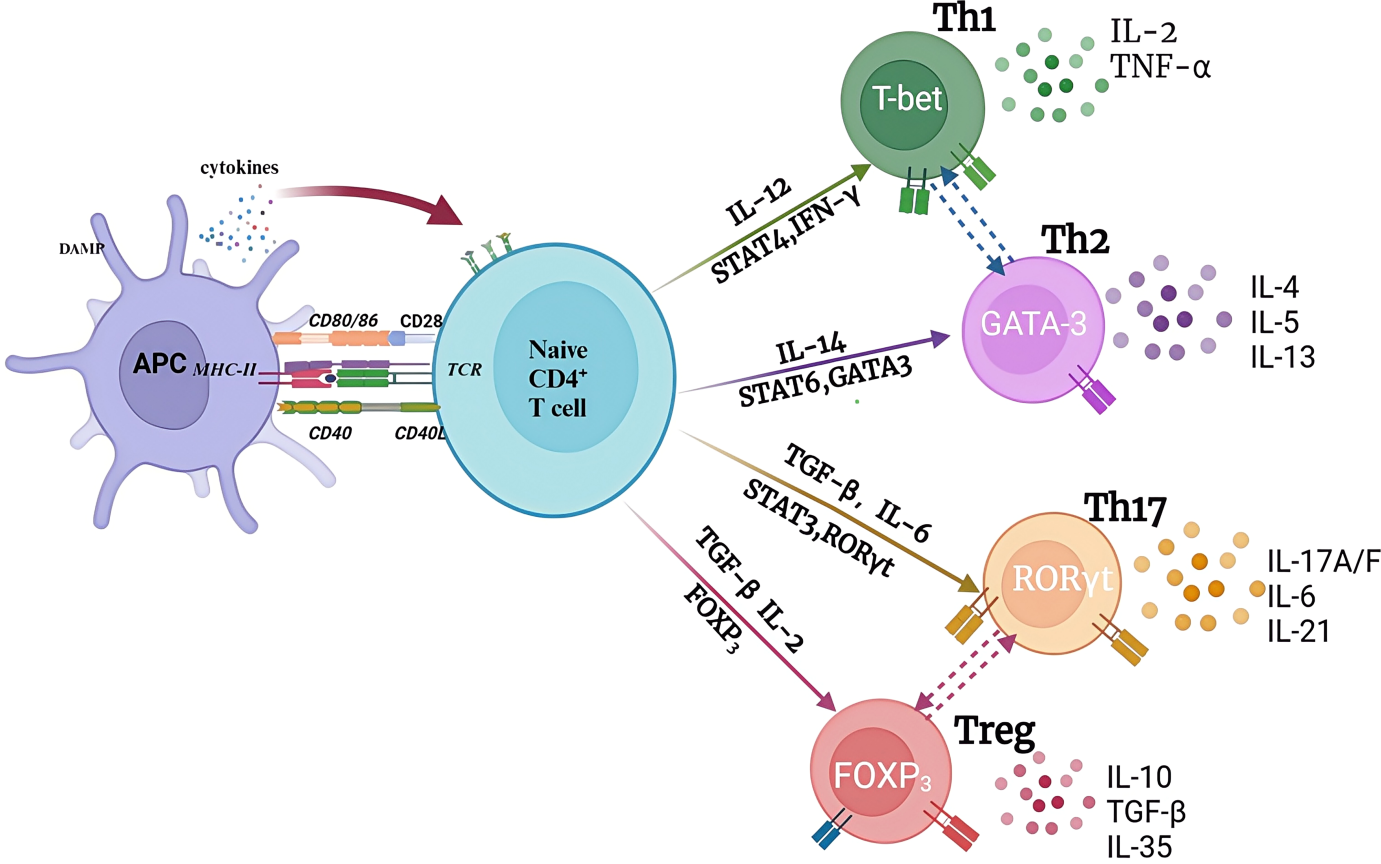
The pathways of naïve CD4^+^ T cells. when encountering with antigens, naive CD4+ T cells are activated and differentiated into different functional subsets under different cytokines stimulation and transcriptional factors regulation in the local microenvironment. Each CD4 subset produces specific cytokines and reaches specific antigen sites to exert unique anti-tumor or anti-inflammatory functions. APC, antigen presenting cell.

CD4-expressing T lymphocytes are also called helper T cells, which are considered to be the main source of cytokines (26). CD4^+^ T cells that were not stimulated by antigen are termed Th0. Stimulation by cytokines and antigens cause Th0 cells to differentiate into various lineages. Pathogens, interleukin (IL)-12, and interferon gamma (IFN-γ) induce Th0 to Th1 differentiation, which activates signal transducer and activator of transcription 4 (STAT4) (27) (**Figure 4**). Common bacteria and soluble antigens, as well as IL-4, can rapidly activate the transcription factor GATA binding protein 3 (GATA3) to induce Th0 to Th2 differentiation. IL-4 is mainly produced by natural killer T (NKT) cells, and eosinophils and basophilic cells in the local environment. T0 cells differentiate into Th17 cells under the influence of IL-6 and transforming growth factor TGF-β, and these cells characteristically produce the cytokines IL-17 and IL-22 and express the master transcription factor retinoic acid (RA)-related orphan receptor γ (RORγt) (28). Th0 cells are induced to differentiate into regulatory T cells (Tregs) under the actions of IL-2 and TGF-β, and Tregs play an important role in immune tolerance and maintenance of immune homeostasis through direct contact with target cells or via cytokine secretions (TGF-β, IL-35, and IL-10) (29).

#### 3.1.1 Th1/Th2 cells

Th1 cells tend to produce proinflammatory responses and secrete Th1-type cytokines, such as tumor necrosis factor (TNF), IL-2, and IFN-γ. These cytokines promote further Th1 proliferation, which affects cellular immunity, and also inhibit Th2 proliferation. Th2 cells secrete IL-6, IL-5, and IL-4 to play an auxiliary role in antibody-producing B cell development. Excess Th2 cells counteract Th1-mediated bactericidal effects. Prolonged redness and swelling are often observed at the site of Bacillus Calmette-Guérin (BCG) vaccination in children suffering from the acute phase of KD. BCG scar activation is indicative of a delayed-type hypersensitivity reaction mediated by Th1 cells (30). Therefore, KD is clinically considered to be skewed towards Th1. However, the functional status of Th1 and Th2 cells remains controversial. Matsubara et al (31) found that during acute phase KD, there was a decrease in the number of CD3^+^ T cells producing IFN-γ, but not in the number of CD3^+^ T cells producing IL-4. It was suggested that in acute KD, there is an imbalance between Th1 and Th2 cells, with Th2 predominating. The transcription factors T-box transcription factor 21 (TBX21, also known as T-bet) and GATA-3 have important functions in Th1 and Th2 subset differentiation, respectively. Studies have found that the levels of mRNA encoding T-bet, GATA-3, and IL-4 in the peripheral blood of patients with KD in the acute phase are reduced significantly, suggesting that Th1 and Th2 cell functions are inhibited at the level of transcriptional regulation, supporting the view that Th1 and Th2 cytokines produced by T cells in KD are functionally inhibited. However, Lee et al. (32) found that in plasma, Th1 (IFN-γ, IL-2) and Th2 (IL-4 and IL-10) cytokine levels were increased significantly in acute phase KD. Thereafter, the plasma levels of these cytokines decreased significantly as the clinical stage progressed. These two groups showed no significant differences, irrespective of a lack of response to initial IVIG therapy or the existence of CALs. This implied that Th1 and Th2 cells might both be activated during the acute phase of KD. Of course, the differences in experimental methods and detection methods might be the main reasons for the differences; however, the results reported to date suggest an unbalanced function of peripheral blood T lymphocytes in acute KD.

#### 3.1.2 Treg/Th17 cells

Tregs mainly expresses cytotoxic T lymphocyte-associated antigen-4 (CTLA-4), CD25, CD4, and forkhead box P3 (Foxp3). Foxp3 is a Treg-specific molecular marker. The CD25^+^CD4^+^ regulatory T cell population plays an important role in maintaining immune tolerance and controlling the antimicrobial immune response (33). In 2004, Furuno et al. found that in the peripheral blood of children with acute stage KD, the abundance of regulatory T cells decreased, as did the expression of CTLA4 and glucocorticoid-induced TNFR-related protein (GITR), which are regulatory molecules related to activation, and IVIG treatment could promote the activation and expansion of Tregs (34). Franco et al. (35) found a subgroup of Tregs that specifically recognized Fc and primarily secreted IL-10, and amplification of these Fc-specific Tregs occurred only in children with coronary artery injury, indicating the importance of Fc-specific Tregs in the prevention of CALs. Moreover, the high miR-223-3p expression in the serum of patients with KD promoted the apoptosis of human coronary artery endothelial cells (HCAECs), suggesting that KD patient serum levels of miR-223-3p might affect HCAEC apoptosis and inflammation by regulating Foxp3 expression, thereby, participating in KD progression.

Th17 cells are pro-inflammatory immune cells capable of secreting the cytokine IL-17. Th17 cells and Tregs are closely related, and under normal circumstances, these two populations maintain a dynamic balance. I T cells can differentiate into induced Tregs (iTregs) in the presence of TGF-β and the absence of IL-6 (36). However, when both TGF-β and IL-6 are present, I T cells will differentiate into Th17 cells. Under physiological conditions, Tregs can inhibit the development of Th17 cells by blocking RORγt expression through the Foxp3-STAT3 signaling pathway. The quantity of Th17 cells and the levels of related cytokines (IL-17, IL-22, and IL-23) increase significantly during the acute phase of KD, and IVIG treatment can reduce their expression. This implies that Th17 functions in KD pathogenesis. Imbalanced Th17/Tregs might be an important factor causing immune dysfunction and leading to IVIG resistance in KD (37). IL-35 is mainly secreted specifically by Tregs, which not only promotes and enhances the expansion and function of Tregs, but also inhibits the differentiation of Th17 cells and reduces the production of IL-17. Sun et al. (38) found that IL-35 levels were lower in patients with KD with CALs, suggesting that IL-35 has an inhibitory effect on acute vasculitis and coronary artery injury. IL-35 promotes Tregs’ immunosuppressive functions via the inhibition of Treg proliferation and transdifferentiation into a Th17-like phenotype, which might protect against KD (39).

### 3.2 CD8^+^T cells

**Figure 5:**
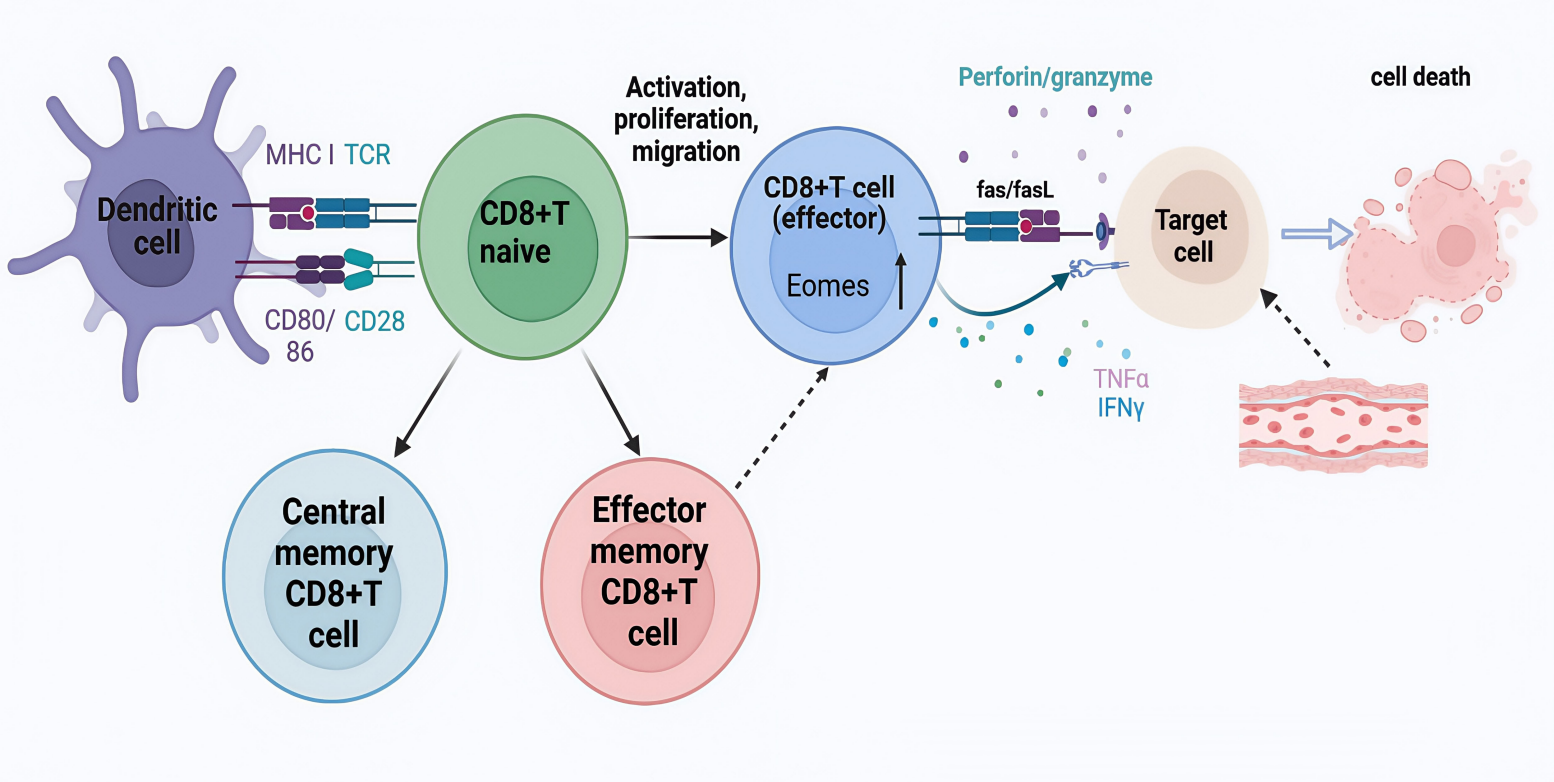
CD8^+^T cell activation and differentiation. Naive CD8+T cells are activated and functionally differentiated upon presentation of cognate antigens by antigen-presenting cells such as dendritic cells (DC) in the presence of costimulation (CD28-CD80/86) and cytokines. Effector CD8+T cells cause injury or death through perforin and granzyme interaction, Fas and Fas ligand (FasL) interaction, and direct or indirect action on target cells by cytokines such as interferon (IFN)-γ and tumor necrosis factor (TNF)-α.

CD8^+^ T cells are a core component in maintaining immune balance and defense mechanisms and its excessive activation and cytotoxic effect are one of the important factors leading to disease progression and vascular injury (40). CD8+T cells are cytotoxic T cells with killing function which bind to major histocompatibility complex class I (MHC I) molecules on their surface and recognize antigen fragments presented on MHC I. Upon recognition of the antigen of the pathogen, CD8+T cells are activated, proliferate and differentiate into cytotoxic T lymphocytes (CTLS) in the peripheral immune organs, and release cytotoxic particles, such as perforin/granzyme, or induce apoptosis through Fas/FasL pathway. In addition, CD8+ T cells can also secrete cytokines, such as interferon (IFN) -γ and tumor necrosis factor α (TNF-α), which further regulate the immune response and enhance the activity of immune cells(**Figure 5**).Brown et al. observed CD8^+^ T and CD4^+^ T cell infiltration in coronary artery aneurysms of eight children who died in the acute phase of KD, in which CD8^+^ T lymphocytes predominated over CD4^+^ T lymphocytes. CD8^+^ T lymphocytes play an important role in the immune response to acute KD, which suggests that major histocompatibility complex class I molecules are involved in the antigen processing of intracellular pathogens, especially viruses, in the pathogenesis of KD (17).

LCWE-induced mouse models can mimic the main pathological features of human KD, including the infiltration of Tregs, and CD4^+^ T and CD8^+^ T cells into CALs, however, CD8^+^ T cells play a key role in the development of KD. Exhaustion of CD8^+^ T cells leads to reduced KD lesion formation and vasculitis. In Rag-1^−/−^ mice (resistant to LCWE-induced KD) the development of vasculitis was promoted by the adoptive transfer of CD8^+^ T cells, including strong infiltration of inflammatory cells into the aorta and coronary arteries. This suggested that targeting CD8^+^ T cells might be used to treat KD (19). Similarly, in comparison with that of healthy controls, patients with KD had a lower proportion of CD8^+^ T cells, especially memory CD8^+^ T cells, in their peripheral blood, suggesting that the proportion of CD8^+^ T cells is an important risk factor for KD. At the same time, single-cell T-cell receptor (TCR) sequencing revealed the specific clonal expansion of CD8^+^ T cells after treatment, indicating that KD is more likely to be triggered by conventional antigens rather than superantigens (41).

### 3.3 CD4^+^/CD8^+^ T cells

The ratio of CD4^+^ T to CD8^+^ T cells is a sensitive index to judge the disorder of human immune function, which can more intuitively reflect the changes in immune function in KD. An increased CD4^+^/CD8^+^ ratio corresponds to enhanced immune function (42). In the acute phase of KD, the abnormal activation of T cells results in abnormal immune system activation and the cascade release of inflammatory factors, which is the key step and initial link of immune system activation. In peripheral blood, the CD4^+^/CD8^+^ T lymphocyte ratio increases in the acute phase, and leading to speculation that an increase in the CD4^+^/CD8^+^ T lymphocyte ratio is related to non-sensitivity to IVIG (43).

## 4 IVIG resistance associated with KD

The first-line treatment for KD is IVIG. In patients with KD, early high-dose IVIG combined with aspirin can reduce the likelihood of them developing CALs; however, 10–20% of patients with KD do not respond to the initial IVIG treatment. IVIG-resistant patients have an increased risk of coronary aneurysm and serious complications, which can endanger the life of children (44). In IVIG-unresponsive pediatric KD patients, cyclosporine can be used as a rescue therapy. Its pharmacological mechanism includes reducing T cell activation and the associated inflammatory response (45).

Ye et al. (46) studied the value of T cell activation in KD diagnosis and the prediction of sensitivity to IVIG, By analyzing CD4^+^ T and CD8^+^ T cells and their surface activation markers (CD69, CD25, and major histocompatibility complex, class II, DR (HLA-DR) in the peripheral blood of children with KD in the acute phase, the authors noted that the over-activation of CD8^+^ T cells was higher than the inhibitory effect of IVIG, leading to autoimmune imbalance, thus the CD3^+^CD8^+^HLA-DR^+^ T lymphocyte to CD3^+^CD8^+^CD69^+^ T lymphocyte ratio (i.e., the late CD8^+^ T cell activation to early CD8^+^ T cell activation ratio) might be a predictor of IVIG-resistant KD.

Interestingly, another study found that the CD8^+^ T lymphocyte-mediated immune response was dominant in children with KD with effective IVIG treatment, and T cell suppression was stronger in non-responders than in responders before IVIG treatment. Moreover, the activation of CD8^+^ T lymphocytes can make KD more sensitive to treatment. Improved suppression of CD8^+^T lymphocytes could increase the therapeutic response (47).

IVIG can effectively target Th17 cells *in vivo* through multiple mechanisms. This includes modulating antigen-presenting cells (APCs), expanding Tregs, and exerting direct effects on Th17 cells. Tregs can negatively regulate the inflammatory response through direct contact with target cells or the secretion of inhibitory cytokines (IL-10 and IL-35). Previous studies have found that the number and function of Tregs are decreased in acute KD, while treatment with IVIG promotes the activation and expansion of Tregs (34, 35, 48). Hirabayashi et al. (49) found that the percentage and absolute number of CD4^+^CD25^+^Foxp3^+^ Tregs in IVIG-resistant children were significantly lower than those in IVIG-sensitive children before IVIG treatment. This suggested that an absence of CD4^+^CD25^+^Foxp3^+^ Tregs before treatment can predict the occurrence of non-response to IVIG in KD. de Groot et al. found that tregitopes within the Fc and F(ab′)2 fragments were identified as regions that bind to multiple human leukocyte antigen (HLA) class II determinants. This binding leads to the expansion and activation of FoxP3+ regulatory T cells (50). Moreover, in the study conducted by Mohan S. Maddur et al., purified CD4^+^T cells were used without any additional accessory immune cells. The researchers demonstrated that the suppression of Th17 cell function by IVIG was mediated through F(ab′)2 fragments. Additionally, IVIG was found to inhibit pSTAT3, a crucial transcription factor involved in the differentiation, amplification, and stabilization of Th17 cells (51). In addition, Tregs require TGF-β for their differentiation and specifically express the transcription factor FoxP3. They also secrete inhibitory cytokines, including IL-10 and TGF-β. M M-H Guo et al. demonstrated that IVIG treatment leads to an increase in the expression of FoxP3 mRNA associated with Tregs, indicating that IVIG exerts its immunosuppressive effects by upregulating Treg cells (52).

There is homology between Th17 and Treg cells, and their balance maintains the stability internal environment of the body. In KD, the Th17/Treg balance is disrupted, which might be an important factor causing immune dysfunction and leading to IVIG non-responsive KD. Th17 can produce IL-17A to IL-17F to protect against IVIG by activating transcription factors CCAAT enhancer binding protein (C/EBP)β and C/EBPδ to induce a hyper-inflammatory cytokine storm, such as granulocyte-macrophage colony-stimulating factor (GM-CSF) and IL-6. Jia et al. (37) found that Th17 increased and IL-17A and IL-6 maintained high levels in KD children in the IVIG non-response group, suggesting that abnormal activation of Th17 and an imbalance of Th17 ratio affecting the body’s immune function were important factors leading to IVIG-resistant KD.

Since the COVID-19 pandemic in 2019, the number of children with KD-like multisystem inflammatory syndrome (MIS-C) has gradually increased (53). MIS-C has attracted the attention of scholars around the world because it has similar clinical symptoms to KD and even meets all the diagnostic criteria for KD. The increase in inflammatory cytokines (IL-6, IL-1β, IL-23, and IL-17) and the expansion of immune cells (Th1, Th2, Th17, and CD8^+^ T cells) occur in the acute phase of KD. T cells play a key role in regulating and coordinating immune responses, and might have a crucial function in KD pathogenesis. Although the exact etiology of KD is still unclear, research on T cells has made progress, and is anticipated to reveal more clues for the diagnosis and treatment of the disease in the future. In the future, in-depth research on T cell subsets, immune regulatory mechanisms, and the development of more precise treatment strategies will help improve the prognosis of patients with KD. Continuing to explore the association between T cells and KD will enhance our understanding of the disease and provide an important scientific basis to improve the prognosis and quality of life of patients.

Cyclosporine is a specific T-cell inhibitor that obstructs the calcium-driven calcineurin-NFAT (nuclear factor of activated T cells) pathway, and it has been explored as a therapeutic option for KD. Several studies have demonstrated that cyclosporine can be effective in treating both KD and cases of resistant KD, suggesting its potential as a valuable treatment alternative (54, 55).

The IL-17 family consists of six structurally related cytokines that signal non-hematopoietic cells to induce innate immune defense mechanisms. IL-17 may contribute to vasculitis by inducing endothelial cells to produce myeloid chemokines (56). Interestingly, IVIG has been shown to inhibit the differentiation of Th17 T cells and suppress the production of IL-17A and IL-17F by T lymphocytes. However, studies have found elevated levels of IL-17A, IL-17C, and IL-17F to be indicative of KD, particularly in patients with coronary artery aneurysms. Notably, IL-17A can effectively differentiate KD from other febrile controls (57). In addition, IL-17A activates nuclear C/EBPβ and C/EBPδ, which diminishes the significant anti-inflammatory effects of IVIG, resulting in a pronounced production of KD-related cytokines, such as G-CSF and IL-6, by human coronary artery endothelial cells, Which indicated that developing agents targeting the activity of C/EBPβ and C/EBPδ could be beneficial for patients with IVIG-resistant KD (58).

## 5 Future therapeutic implications

T cell activation plays a crucial role in the progression of KD, making T cell biomarkers valuable for clinical diagnosis. These markers can provide predictive insights into the disease’s development and potentially assist in tailoring treatment strategies for affected patients.The percentage of CD8^+^ HLA^-^DR^+^ T cells can serve as a diagnostic indicator for KD. While IVIG suppresses the activation of CD8^+^T cells, excessively high levels of CD8^+^T cell activation may lead to IVIG resistance. Additionally, the ratio of CD3^+^ CD8^+^HLA^-^DR^+^ T cells to CD3^+^ CD8^+^ CD69^+^ T cells can be used as a predictive marker for IVIG sensitivity (46). Research has found that elevated serum levels of IL-17A before and after intervention are associated with IVIG resistance in KD patients. A serum IL-17A level of ≥39.96 pg/mL is promising as a predictive biomarker for IVIG-resistant KD. Furthermore, combining the assessment of IL-17A with CRP, WBC count, and ALT levels can enhance the accuracy of diagnosing IVIG-resistant KD (59). Moreover, the IVIG-resistant group continued to exhibit significantly higher levels of IL-17A and IL-6, suggesting that the aberrant activation of Th17 cells may contribute to IVIG-resistant KD through their inflammatory characteristics. Additionally, elevated concentrations of IL-6 and IL-23 were observed in KD patients, indicating an imbalance between Th17 and Tregs in those with acute KD (37). Li et al. found that elevated levels of circulating IL-35 enhance the immunosuppressive functions of Tregs by inhibiting cellular proliferation and promoting the transdifferentiation of Tregs into a Th17-like phenotype. This suggests a protective mechanism associated with KD. Therefore, IL-35 and its derivatives may hold therapeutic potential for the treatment of KD (39).

As for clinical trails targeting to T cells in KD, J.C. Burus et al. demonstrated acute inflammation, including myocarditis, was found to be associated with a significant increase in circulating myeloid dendritic cells in older children with acute KD. A low number of CD4^+^ILT4^+^tolerogenic myeloid dendritic cells was correlated with lymphadenopathy as an initial clinical manifestation. Furthermore, increases in CD4^+^ and CD8^+^ T lymphocyte counts and lymphocyte reductions were not associated with any specific patterns within the innate population (60). Yu Hirabayashi et al. found that the percentage and absolute numbers of CD4^+^CD25^+^FOXP3^+^Tregs were significantly reduced in patients during the acute phase of KD, but markedly increased following IVIG treatment (49). Song et al. discovered that CD14 + monocytes play a pivotal role in the cell communication processes during the activation of vascular inflammation in KD and identified SELPLG and ITK as important signaling genes involved in this communication (61).

Recent advances in single-cell RNA sequencing (scRNA-seq) and spatial transcriptomics have revolutionized the study of immune dysregulation in inflammatory diseases. However, their application to KD, particularly in elucidating the heterogeneity of T-cell subsets (e.g., Th17, Tregs, cytotoxic CD8^+^T cells) during acute and convalescent phases remains underexplored. The integration of transcriptomics, proteomics, and metabolomics could provide a more comprehensive view of KD immunopathogenesis. Despite several studies performing extensive transcriptomic analyses of peripheral blood and discovering dysregulation of the innate immune system, potentially caused by the hyperreactivity of neutrophils. Research conducted by Wang et al. observed significant temporal changes in cell abundance before and after IVIG treatment through single-cell sequencing of peripheral blood. These findings align closely with previous studies. Notably, the abundance of B cells, CD8^+^ T cells, and NK cells may help differentiate KD from other febrile illnesses and could serve as predictors of IVIG treatment response (41). In the study by Fan et al, an analysis of DEGs in immune cells from children with KD compared to healthy controls revealed that the dominant cell clusters in healthy children included NKT cells, CD4^+^ cytotoxic T cells, T helper cells, CD8+ memory T cells, and naive CD8^+^ T cells. The researchers identified three DEGs common to these T cell clusters: IL7R, CD3D, and CD27. IL7R encodes the interleukin 7 receptor, which is a critical marker for T cell development and plays an important role in immune function. CD3D is associated with immune checkpoints and may provide a novel therapeutic target for KD. Meanwhile, CD27 is a marker for memory B cells and is also detected in normal plasma cells; it is believed that CD27+ memory B cells contribute to the pathogenesis of inflammation in KD (62). Geng et al. identified a Kawasaki Disease-related classical monocytes (KD-CM) associated with neutrophil activation using scRNA-seq analysis. The marker genes of KD-CM are primarily linked to neutrophil activation, suggesting that KD-CM may regulate vascular inflammation in KD by stimulating neutrophils (63).

Alex Kentsis et al. analyzed the urinary proteomes of patients diagnosed with KD compared to those initially suspected of having KD but ultimately diagnosed with other febrile illnesses. They identified over 190 candidate biomarkers for KD. These molecules include potential markers of endothelial and myocardial injury (such as talin, filamin, desmoglein, obscurin, and titin), indicators of leukocyte activation (including AMICA1, CAECAM, CXCL12, GDF15, and LAIR1), markers involved in pathogen immune recognition (DMBT1, ABCB9), and molecules related to cytokine regulation (CSMD3, meprin A) (64). In addition, Yayoi Kimura et al. identified significantly elevated levels of LBP and LRG1 in the serum of patients with acute KD. LBP and LRG1 may serve as useful biomarkers for monitoring the acute phase of KD in a clinical setting. Additionally, RPB4 represents a promising potential biomarker for tracking disease progression and treatment efficacy (65). Ho-Chang Kuo et al. conducted a plasma proteomics analysis between four groups that are KD patients before intravenous immunoglobulin treatment, at least three weeks after treatment, non-fever control, and fever control children. and identified ZnuC as an ideal target for the generation of specific antibodies in children with KD (66).

KD is a systemic immune vasculitis that commonly leads to acquired heart disease in children, characterized primarily by persistent fever. Although the exact etiology of KD is not fully understood, it is posited to arise from an exacerbated inflammatory response triggered by both infectious agents and genetic predispositions. This review emphasizes the pivotal role of T lymphocytes in the pathogenesis of KD, particularly highlighting the dynamics of CD8^+^ T cell expansion and the dysregulation of Th17 cells and Tregs. Such imbalances are implicated in the phenomenon of IVIG resistance and ongoing inflammation in affected patients. Our review indicates that therapeutic strategies aimed at modulating T cell activity may hold promise for enhancing clinical outcomes in KD. Specifically, we propose exploring the potential of innovative immunomodulatory agents, including cyclosporine and inhibitors and targeting IL-17 and IL-23, as avenues for novel treatment approaches. This review not only underscores the critical immunological factors contributing to KD but also offers insights into future therapeutic directions that could improve management and outcomes for children suffering from this complex disease.
